# Notes and News

**Published:** 1901-08-24

**Authors:** 


### NOTES AND NEWS.

Mr. Peregrine Purvis has made a donation of £1,000 to the Royal Free Hospital, Gray's Inn Road.

There are still a few cases of plague in Egypt distributed over a large area. The mortality has been nearly 50 per cent. throughout the visitation. There are now a few cases in Hong Kong, which a little time back was declared to be free from the disease.

The Alfred Bevan Convalescent Home for Men, Women and Children has been opened at Sandgate. It is a working man's project on the same lines as the Morley Home at St. Margaret's Bay. The late Sir Alfred Bevan contributed £4,500 to the scheme, hence the title of the Home. The institution contains 250 beds.

Boards of Guardians have often proved the greatest enemies to vaccination. They have until now had it in their power to impede the action of the vaccination officer, and have in some cases practically prevented prosecutions under the Act, with the result that the great army of the unvaccinated has been peacefully growing for some time. Great indignation has recently been aroused among the anti-vaccinators at the action of the Shoreditch vaccination officer, who has brought a good many objectors before the magistrates. The new regulations make the officer independent of the control of the Guardians in this respect.

A scheme to improve the Taunton and Somerset Hospital has been laid before the governors and subscribers of the institution. This includes the enlargement of wards, the remodelling of offices, the provision of new kitchens and equipment, a new lift, fire escape, and heating apparatus. Reflooring and renewal of woodwork and other structural renovations complete a list of requirements which show that the hospital is at present in a very imperfect condition. When so much has to be done it is always a question whether an entirely new building is not the wisest course in the end—that is to say, of course, as far as the original buildings are concerned. The alterations are estimated to cost £6,500, towards which some £3,000 has been promised.

The Archbishop of York opened a new wing at the Scarborough Hospital last week.

The next Congress and Exhibition of the Sanitary Institute will be held at Manchester during September under the presidency of Earl Egerton of Tatton.

The next meeting of the British Medical Association will be held in Manchester under the presidency of Mr Walter Whitehead.

The late Mr. A. Dettmar, of Gloucester Place, Portman Square, has bequeathed to the National Hospital for the Paralysed and Epileptic, Queen's Square, the Hospital for Epilepsy and Paralysis, in Regent's Park, and St. Mary's Hospital, a yearly sum of money for 15 years; the exact amount is to be decided by the trustees under the will. Later, the capital will be divided amongst the three hospitals.

Dr. Legge, Medical Inspector of Factories, states that the certifying surgeons under the Factory Acts will this year receive such portions of the annual report of the Chief Factory Inspector of Factories as have medical interest. This it is hoped will show them the importance attached to their work, and the use to which their reports are put.

The trustees under the will of the late Marquis of Bute are proving very liberal in respect to the proposed Seamen's Hospital at Cardiff. They have already promised to provide the same amount which has been contributed by the public, namely, £13,242. This will make a sum of nearly £26,500, leaving only £3,500 to be collected to complete the estimated requirement of £30,000. Beyond this it is generally understood that the present Marquis will grant the site which the late Marquis intended for the purpose.

In 1903 the International Congress of Medicine is to be held at Madrid. Unless the present sanitation of the city is altered the medical visitors will have opportunity to study what to avoid. The Government Board of Health has just published statistics revealing an alarming death rate. The figures relating to infant mortality are especially significant. They form nearly 50 per cent. of the whole death rate of the population. Yet typhoid plays a very insignificant part in this record of disease.

There is no profession which has so much demanded of it as the medical profession. A medical education is an expensive one, spread as it is over a large number of years, and in the face of much competition most young general practitioners find it hard to gain a living. And yet individuals and public bodies alike demand gratuitous services of medical men without the least scruple. A case in point has recently come to our notice at Halifax. The Halifax Guardians for many years past have employed the services of Dr. Dolan, as Medical Officer at the workhouse. Now the Guardians have a Poor Law Union Hospital at Salterhebble. They decided that a medical officer should be engaged for Salterhebble, and they offered the post to Dr. Dolan, the salary (if any) to be decided later on. He, however, refused the post without salary. Now the Guardians are advertising for a medical officer for the hospital at a salary of 100 guineas.

